# A retrospective cohort study of 27,049 polytraumatized patients age 60 and above: identifying changes over 16 years

**DOI:** 10.1007/s41999-021-00546-9

**Published:** 2021-07-29

**Authors:** Y. Kalbas, M. Lempert, F. Ziegenhain, J. Scherer, V. Neuhaus, R. Lefering, M. Teuben, K. Sprengel, H. C. Pape, Kai Oliver Jensen, The TraumaRegister DGU

**Affiliations:** 1 Department of Trauma, University Hospital Zurich, Raemistrasse 100, 8091 Zurich, Switzerland; 2 Institute for Research in Operative Medicine (IFOM), University of Witten/Herdecke, Cologne, Germany; 3 Committee on Emergency Medicine, Intensive Care and Trauma Management (Sektion NIS) of the German Trauma Society (DGU), Berlin, Germany

**Keywords:** Geriatric trauma, Interdisciplinary, Polytrauma, Epidemiology, Changes, Outcome

## Abstract

**Purpose:**

The number of severely injured patients exceeding the age of 60 has shown a steep increase within the last decades. These patients present with numerous co-morbidities, polypharmacy, and increased frailty requiring an adjusted treatment approach. In this study, we establish an overview of changes we observed in demographics of older severe trauma patients from 2002 to 2017.

**Methods:**

A descriptive analysis of the data from the TraumaRegister DGU^®^ (TR-DGU) was performed. Patients admitted to a level one trauma center in Germany, Austria and Switzerland between 2002 and 2017, aged 60 years or older and with an injury severity score (ISS) over 15 were included. Patients were stratified into subgroups based on the admission: 2002–2005 (1), 2006–2009 (2), 2010–2013 (3) and 2014–2017 (4). Trauma and patient characteristics, diagnostics, treatment and outcome were compared.

**Results:**

In total 27,049 patients with an average age of 73.9 years met the inclusion criteria. The majority were males (64%), and the mean ISS was 27.4. The proportion of patients 60 years or older [(23% (1) to 40% (4)] rose considerably over time. Trauma mechanisms changed over time and more specifically low falls (< 3 m) rose from 17.6% (1) to 40.1% (4). Altered injury patterns were also identified. Length-of-stay decreased from 28.9 (1) to 19.5 days (4) and the length-of-stay on ICU decreased from 17.1 (1) to 12.7 days (4). Mortality decreased from 40.5% (1) to 31.8% (4).

**Conclusion:**

Length of stay and mortality decreased despite an increase in patient age. We ascribe this observation mainly to increased use of diagnostic tools, improved treatment algorithms, and the implementation of specialized trauma centers for older patients allowing interdisciplinary care.

## Key summary points

**Aim:**

In this study, we establish an overview of changes we observed in demographics of older severe trauma patients from 2002 to 2017.

**Findings:**

Trauma mechanism, as well as injury pattern, changed over time. We found length of stay and mortality decreased despite an increase in patient age.

**Message:**

We ascribe this observation mainly to increased use of diagnostic tools and improved treatment algorithms and underline the importance of the implementation of specialized geriatric trauma centers allowing interdisciplinary care.

## Introduction

Western European society is aging with ubiquitous demographic changes. In Switzerland, an increase of people aged over 65 years is expected: from 29.1% of the population in 2015 up to 48.1% in 2045 [1]. A similar trend can be observed in Germany, where the Federal Bureau of Statistics (“Statistisches Bundesamt”) predicts the number of inhabitants older than 67 years of age to surpass 21 million in the year 2039, constituting an increase of about 6 million [2]. As a direct consequence, a shift towards a growing number of older (trauma) patients is expected [3]. Recently, the increasing relevance of trauma care for older patients has also been demonstrated by epidemiological studies [4] reporting that patients over 65 years of age account for 23% of all trauma admissions. In addition, the increasingly active lifestyles in older patients contribute to higher incidences of severely injured cases in this patient group, a trend that is expected to continue in the following decades [4–6]. Moreover, trauma is the fifth most common cause of death in older patients [7, 8].

These developments will pose a challenge for trauma care providers all over the world since advanced biological age has been identified as an individual risk factor for negative outcomes in trauma [9]. Several groups reported mortality rates to be three to six times higher in older patients compared to younger counterparts [10, 11]. Providing trauma care to older patients is particularly challenging and complex as they often present in a frail state, making them more prone to complications, regardless of injury severity [12–15]. To optimize trauma care for the group of older patients, current strategies may require tailored adjustments based on their special needs. Older patients require an even more rapid, yet prudent and farsighted, treatment to reduce morbidity and mortality [4, 6].

The aim of this study was to investigate demographics, diagnoses, treatments, and outcomes in older (60 + years) polytraumatized patients over a period of 16 years. Moreover, we examined the differences in a subgroup of even older individuals (octogenarians and older).

## Patients and methods

The present study is in line with the publication guidelines of the TR-DGU and registered as project ID 2018-008.

### The TraumaRegister DGU^®^ (TR-DGU)

The TraumaRegister DGU^®^ of the German Trauma Society (Deutsche Gesellschaft für Unfallchirurgie, DGU) was founded in 1993. The aim of this multi-center database is a pseudonymized and standardized documentation of severely injured patients.

Data are collected prospectively in four consecutive time phases from the site of the accident until discharge from hospital: (A) pre-hospital phase, (B) emergency room and initial surgery, (C) intensive care unit and (D) discharge. The documentation includes detailed information on demographics, injury pattern, comorbidities, pre- and in-hospital management, course on intensive care unit, relevant laboratory findings including data on transfusion and outcome of each individual. The inclusion criterion is admission to hospital via emergency room with subsequent ICU/ICM care or reach the hospital with vital signs and die before admission to ICU. The infrastructure for documentation, data management, and data analysis is provided by AUC—Academy for Trauma Surgery (AUC—Akademie der Unfallchirurgie GmbH), a company affiliated to the German Trauma Society. The scientific leadership is provided by the Committee on Emergency Medicine, Intensive Care and Trauma Management (Sektion NIS) of the German Trauma Society. The participating hospitals submit their data pseudonymized into a central database via a web-based application. Scientific data analysis is approved according to a peer-review procedure laid down in the publication guideline of TraumaRegister DGU^®^.

The participating hospitals are primarily located in Germany (90%), but a rising number of hospitals of other countries contribute data as well (at the moment from Austria, Belgium, China, Finland, Luxembourg, Slovenia, Switzerland, The Netherlands, and the United Arab Emirates). Currently, approx. 30,000 cases from more than 650 hospitals are entered into the database per year.

Participation in TraumaRegister DGU^®^ is voluntary. For hospitals associated with TraumaNetzwerk DGU^®^, however, the entry of at least a basic data set is obligatory for reasons of quality assurance.

### Injury severity score (ISS)

The injury severity score is a scalar measure (1–75) of anatomic injury. It takes sum of squares of the Abbreviated Injury Scale (AIS) grade in the three most severely injured body regions [16, 17]. Body regions are divided into head and neck, face, thorax, abdomen, extremities and pelvis, and external/soft tissues. Injury severity is stratified into minor (1), moderate (2), serious (3), severe (4), critical (5) and maximal (6). Having a maximal injury in one body region yields an instant ISS of 75. While newer, more patient specific, definitions have been proposed [18], an ISS of ≥ 16 is still a very commonly used, accessible and widely available definition of a polytrauma [17].

### Inclusion/exclusion criteria

Patients documented in the TraumaRegister DGU^®^ were included if the ISS was 16 points or higher and the age was 60 years or above. Furthermore, only patients primary admitted to a level one (supra-regional) trauma center in Germany, Austria, or Switzerland between 01/2002 and 12/2017 were considered. Patients transferred out to another hospital within 48 h (1.1% of cases) were excluded due to missing outcome.

### Analysis

Besides the total population of patients 60 years and older, we further analyzed patients 80 years or older separately. Patients were grouped into four phases based on the year of hospital admission. The time phases covered 4 years each: phase 1: 2002–2004; phase 2: 2005–2009; phase 3: 2010–2013; and phase 4: 2014–2017.

Descriptive analysis was performed with mean and standard deviation (SD) for continuous measurements, and number of patients with percentages for categorical variables. A formal test-statistical comparison of the four time periods was avoided since the huge sample size would reveal formal significance even in case of minor non-relevant differences.

## Results

### Demographics

A total of 95,829 patients were documented in the TR-DGU between January 2002 and December 2017. Out of these patients, 79,321 had an ISS of 16 or higher and were primarily admitted to a German, Swiss or Austrian level one trauma center. 27,049 of them (34.1%) were 60 years old or older and thus included (Table 1). In this group, the average age on admission was 73.9 years (SD 8.7), and average age increased over time from 71.7 (8.3) in 2002 to 74.5 (8.9) in 2017. The percentage of people aged 60 + years meeting the inclusion criteria showed a steep increase from 23.0% (phase 1) to 39.5% (phase 4). The portion of females was higher in older patients (36.2%) as compared to patients below the age of 60 (24.1%). Within older patients, the portion of females did not show a trend over time. Mean ISS was about the same in older patients (27.4, SD 11.6) when compared to younger trauma victims (28.2, SD 12.1). In older patients, a slight decrease over time: 28.9 in phase 1, and 26.7 in phase 4 can be notified.

**Table 1:** Prevalence of severely injured older patients in the four time phases

Year	All patients	Patients 60 +-year-olds	Patients 80 +-year-olds
2002–2005	5458	1257 (23.0%)	229 (4.2%)
2006–2009	12,420	3262 (26.3%)	773 (6.2%)
2010–2013	26,187	8600 (32.8%)	2363 (9.0%)
2014–2017	35,265	13,930 (39.5%)	4263 (12.1%)
Total	79,321	27,049 (34.1%)	7628 (9.6%)

Only primary admitted patients with ISS 16 + treated in a level-one trauma center were considered

### Patient and trauma characteristics

In addition to increased aging of the trauma population, a striking increase in the percentage of octogenarians over time was encountered [from 4.2% (phase 1) to 12.1% (phase 4)].

**Fig. 1:**
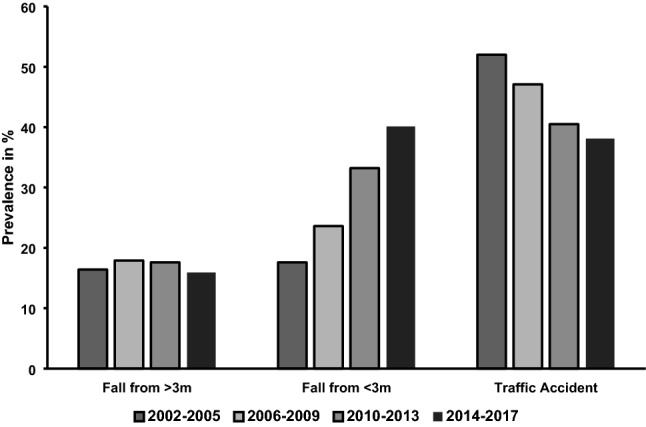
Trauma mechanism

**Fig. 2:**
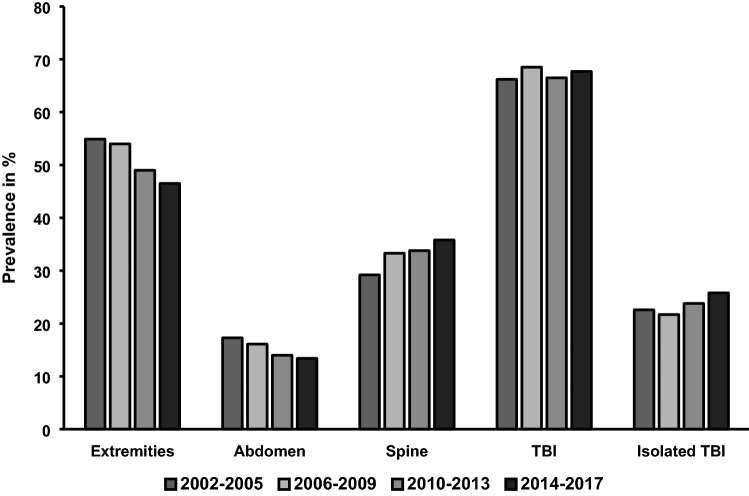
Injury patterns. *TBI* traumatic brain injury

Figure 1 illustrates alterations in documented trauma mechanisms, demonstrating an increase in low falls (< 3 m): 17.6% (phase 1) to 40.1% (phase 4) and drop in the prevalence of traffic injuries: 52.0% (phase 1) to 38.1% (phase 4). The number of falls from higher than 3 m was nearly unaltered (16.4% in phase 1 vs. 15.9% in phase 4). In Fig. 2, alterations in documented injury patterns are displayed: over time a decrease in extremity injuries (54.9–46.5%) as well as abdominal trauma (17.3–13.4%) could be observed, while an increase in spinal injuries (29.2–35.8%) and isolated traumatic brain injuries (22.6–25.8%) occurred. The percentage of traumatic brain injuries in total did not differ relevantly between the phases (range 66.5–68.5%).

**Fig. 3:**
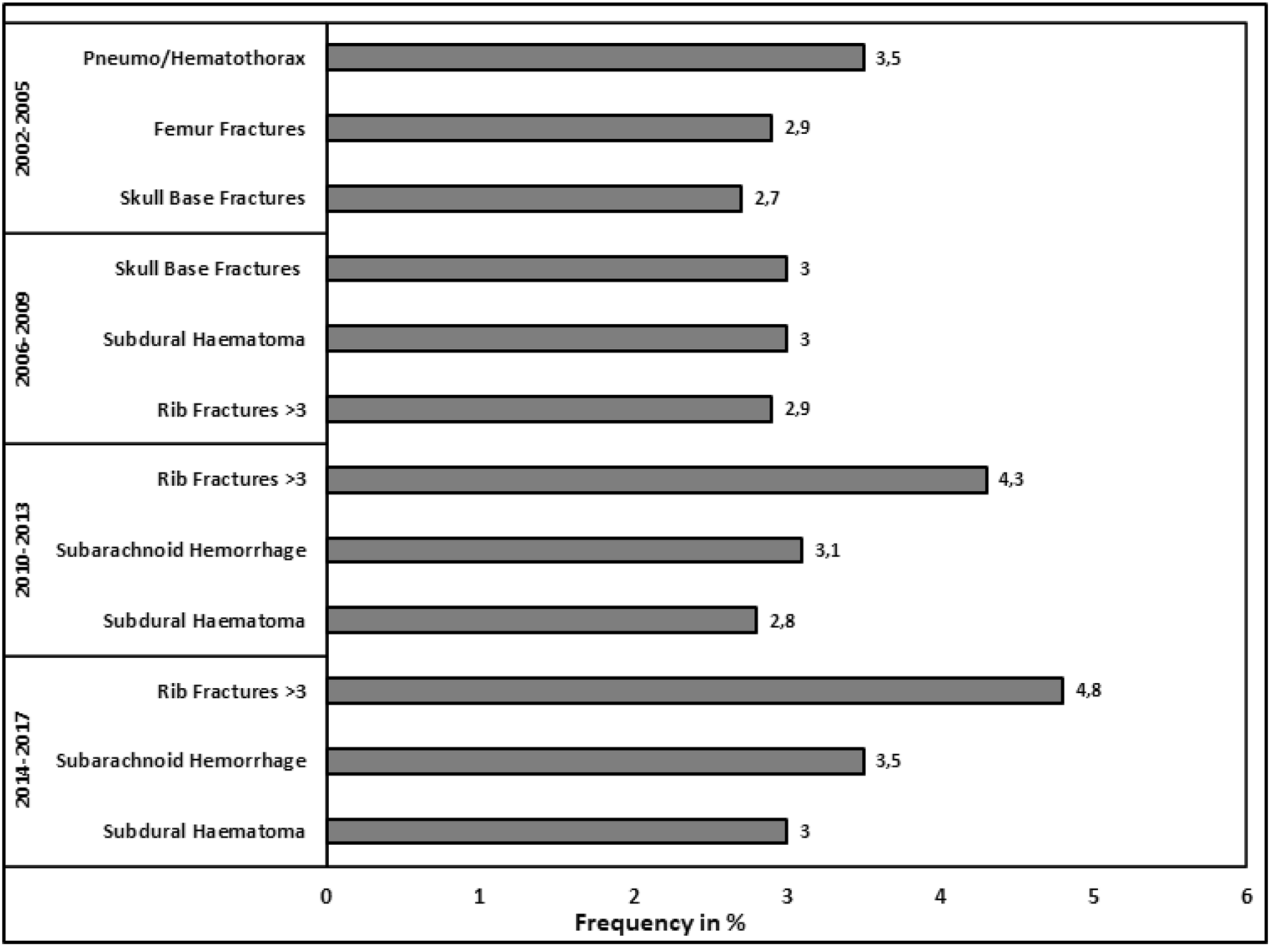
Three most common diagnoses sorted by groups

When comparing the most frequent diagnoses in older patients within the respective groups, we see a clear increase of intracranial hemorrhage over time, while femur fractures decreased. Furthermore, an increase in rib fractures was found (Fig. 3).

### Imaging, therapy, and outcome

**Fig. 4:**
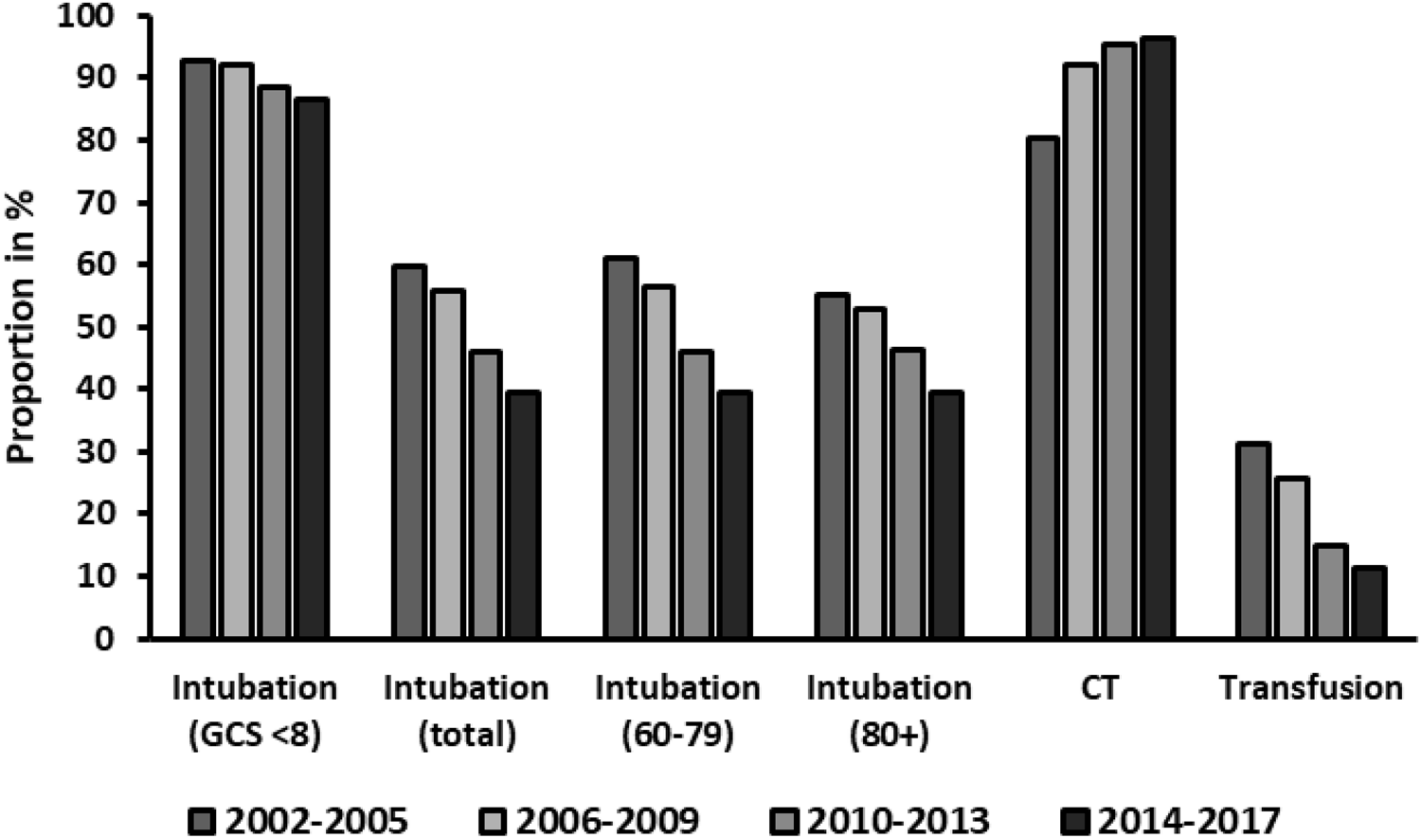
Diagnostic tools and interventions. *CT* computed tomography, *GCS* Glasgow Coma Scale

Figure 4 illustrates the use of diagnostic tools and interventions. As it is clearly shown, the utilization of computed tomography (CT)-imaging increased from 80.2% in phase 1 to 96.3% in phase 4. Intubation rates decreased from 59.8% (1) to 39.5% (4). Moreover, lower in-field-intubation in unconscious patients (GCS ≤ 8) was seen over time (92.8% in phase 1 to 86.5% in phase 4). Additionally, the utilization of blood transfusion was found diminished at the end of the study period. While 31.1% of patients in phase 1 received a transfusion, only 11.8% in phase 4 did.

**Fig. 5:**
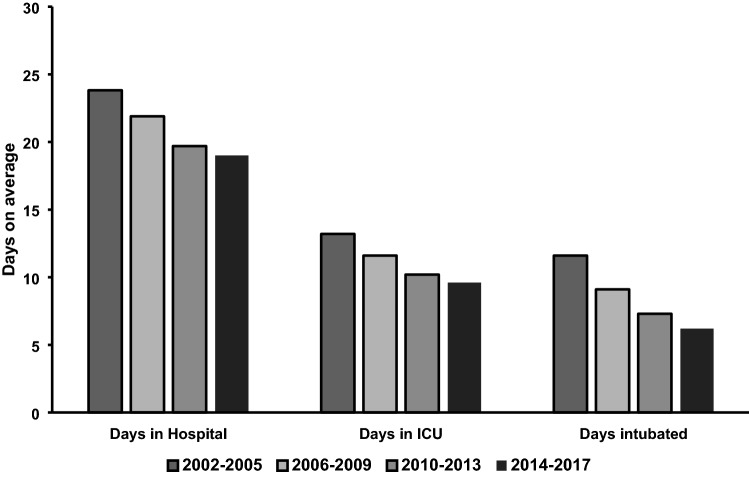
Length of stay. *ICU* intensive care unit

**Fig. 6:**
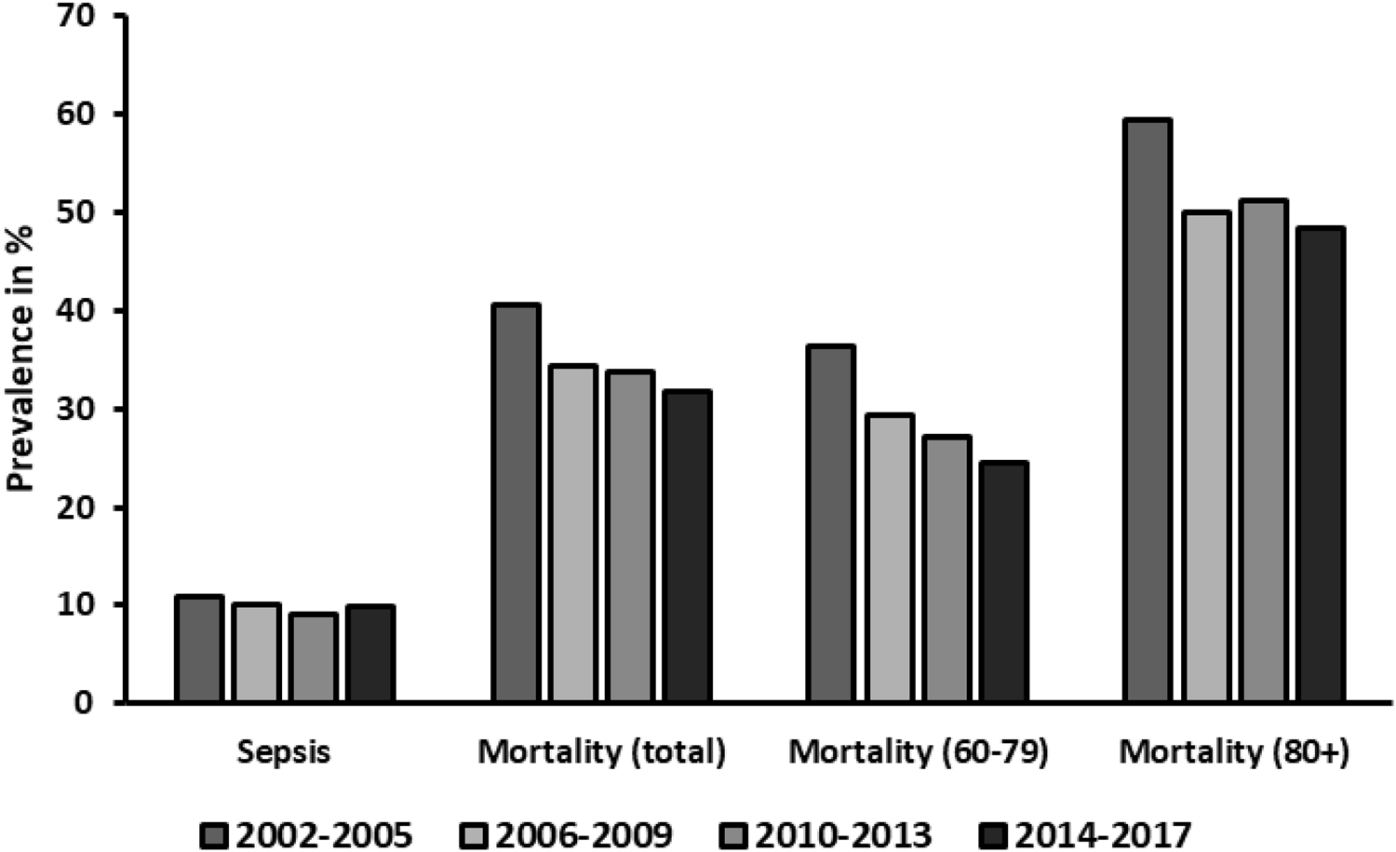
Outcome and complications

Figures 5 and 6 illustrate changes regarding the outcome. Within the entire observation period, average length of hospital stay was found to be shortened from 23.8 (median 15) days in phase 1 to 19 (median 14) days in phase 4, while the average length of stay in the intensive care unit dropped from 13.2 (median 6) days in phase 1 to 9.6 (median 4) days phase 4. Patients were extubated on average 4.2 days earlier (9.6 (median 3) intubated days in phase 1 to 5.4 (median 1) intubated days in phase 4). Moreover, overall mortality decreased from 40.5% (phase 1) to 31.8% (phase 4). When breaking down these numbers into patients under and over the age of 80 years, we see similar trends: While the mortality in 60–80-year-olds decreased from 36.3 to 24.5% over the years, the octogenarians showed a reduction from 59.4 to 48.4%.

The percentage of patients diagnosed with sepsis remained similar (10.8% in phase 1 to 9.8% in phase 4).

## Discussion

Throughout the observation period, we noticed the following trends:

1. Older patients constitute a higher percentage of severely injured patients and include increasing numbers of very old (+ 80 years) patients.
2. Low falls become relatively more frequent and injury patterns change concomitantly with the aging trauma population.
3. Increasing numbers of older severe trauma patients over time show decreased hospitalization times and improved mortality rates.
4. Treatment algorithms change over time leading to an increased use of CT-imaging, a decreased use of blood transfusions and a decreased rate of intubation.

As anticipated, the current study confirms the general perception that the amount of polytraumatized patients over the age of 60 is growing. Simultaneously, the average age of these patients is increasing as well. Our findings exceed the demographic development by a significant amount (16.5% vs. 3.5% increase in people age 60 + from 2002 to 2017 [19]). These results match well with published literature, describing a disproportionately steep increase in trauma admissions amongst older people [8, 20–22]. A possible reason might be an increasingly active lifestyle, the increased use of CT-imaging, and changes in assessment and expectations regarding falls in nursing homes [22]. As a result, the overall trauma population presents with increasing numbers of pre-existing co-morbidities such as cardiovascular and pulmonary diseases, neurological and cognitive deficits, malnutrition, osteoporosis, electrolyte imbalances and polypharmacy [23]. Thereby, the risk for developing complications after trauma increases. The most common complications in older patients are delirium and infection, mostly of the urinary tract and the respiratory system [23]. Understandably, these patients require an increased level of care and a multidisciplinary treatment approach.

Incidences of specific injury mechanisms have changed over time: the rate of low falls (< 3 m) has significantly increased and became the leading mechanism of injury, while the relative number of vehicle accidents has been reduced. Our observations are in line with other epidemiological studies on trauma in older patients [8, 20, 24]. One can assume that the reduction of road traffic accidents (RTAs) has to do with continuous improvements in pedestrian and driver safety due to infrastructural and technical changes [25]. The increased occurrence of falls from less than 3 m coincides with the findings of other groups postulating an increasingly active and independent lifestyle of older people leading to more injury among them [4, 9, 26]. A study performed by Bonne et al. even showed that falls from standing might account for almost 90% of all cases of this trauma mechanism [4]. These changes in trauma mechanisms account for the observed changes to the injury patterns.

Head injuries or traumatic brain injuries (TBI) remain a major concern in trauma care for older patients. With the increase of falls from standing height, the occurrence of isolated head injuries increased as well. People aged 75 years or older are at the highest risk of hospitalization or death related to TBI throughout all age groups [26] with old age being a linear indicator for adverse outcome after TBI [27, 28]. Old patients with TBI may present with only milder trauma mechanisms and without clear neurological deficits and may yet develop relevant intracranial hematoma [29] leading to delays in diagnosis and poor outcome [26]. This situation is further complicated by the increasing use of anticoagulants such as vitamin-k antagonists and other, with recent studies reporting in an increase of prescriptions of 40% within 6 years [30].

The observed increase of spine injuries has also been described by other studies. With older people being more at risk of sustaining spine injuries even from minor trauma [31] and the already discussed general increase of admissions [8] the increasing numbers of older patients presenting with spine injuries is natural. Alternatively, this observation may also be explained by increased use and higher quality of CT diagnostics, both in the trauma bay as well as in the emergency department. This comes with an increased sensitivity for cervical-spine injuries over conventional radiography especially in older patients [32]. As more patients are screened using CT scans, an increasing number of injuries is noted, that might otherwise have been missed or diagnosed with a delay.

It has been suggested by others that the decrease of diagnosed injuries to the extremities and the abdomen, most likely relates to the changes in trauma mechanisms [33], more specifically the decreasing incidences of high-energy trauma such as RTAs. This might be due to better road traffic safety and optimization of car safety [34].

We saw a steep increase in the use of CT diagnostics. Chang et al. reported older patients to be at a significantly higher risk of being under-triaged and under-diagnosed, leading to worse outcomes [35]. By increased use of diagnostic tools such as CT scans, fewer injuries are missed, and the outcome can be improved.

Moreover, we demonstrated that ventilation days and subsequent duration of ICU stay decreased over time. This can be attributed to better treatment regimen tailored to the needs of older trauma patients such as implementing improved standard operating procedures [36]. It seems remarkable, that ISS rates remain unaltered although trauma mechanisms shift towards low energy trauma. Recent studies showed prominent improvements in overall outcome of older trauma patients for centers with designated teams for this specific cohort, compared with general centers [37]. Mangram et al. demonstrated better morbidity and mortality after the implementation of a designated geriatric trauma unit. In addition, time of stay at ICU, ventilation time and the overall length of stay were significantly reduced as well [38, 39]. The implementation of designated trauma teams has proven to decrease the risk of missed injuries, thereby minimizing diagnosis-related complications [40]. Furthermore, the introduction of routine geriatric consultations has led to an improved outcome as demonstrated in recent studies [41, 42]. Combined, these findings suggest the improved standard of care plays a superordinate role in the outcome than the nature of injury (increasing low falls, reduced RTAs).

The observed reduction of provided blood transfusions in older patients can be explained by the increasing implementation of more restrictive transfusion protocols in general in major trauma centers [43–45]. Restrictive guidelines lower the incidence of transfusion-related complications and this trend may have added to the increased survival rates over time [36, 46]. Finally, we noted a decrease in intubation rates, including intubation rates on scene due to neurologically impaired patients. We believe this to be due to a change in paradigms and guidelines for first responders. Studies have shown that early intubation does not improve outcome [47] and should be avoided if possible. The German S3 guidelines for the treatment of polytraumatized patients [48], however, still lists intubation as a soft recommendation. Considering the improved overall outcome and the reduction in days intubated and days spent in the ICU, one can interpret the restrictive use of intubation as having a positive impact.

Finally, when evaluating hospitalization times and mortality, we noted a steep decrease in both. This constitutes an improvement in overall outcome, despite the upper mentioned increase in age and concomitant co-morbidities. Comparing the 60–80-year-olds to the octogenarians, similar trends can be noted. While 60–80-year-olds are still part of the more active older population, octogenarians can be considered more frail and therefore more likely to fall from standing height. Having similar reduction in mortality in both groups suggests that the improved outcomes are not merely due to the population’s composition but that it relates to optimization of treatment algorithms and increased utilization of diagnostic tools. This is further corroborated by the fact that both groups are treated similarly as exemplified by the percentage of intubations.

Overall, our findings match well with previous studies that were performed at a single large level one Trauma center in the TraumaNetzwerk DGU^®^: these studies showed very similar trends concerning admissions, injury patterns, and outcome [21, 49].

## Limitations

As a registry study, the retrospective character is the most important limitation. The TR-DGU has evolved over time, and in 2002, mostly large (level 1) trauma centers participated. During the recent years, registry participation became part of a certification process, and since about 2012 there is a > 90% coverage at least in Germany. One can argue that this comes with a certain bias, as the inclusion of smaller trauma centers could also lead to the inclusion of less severe trauma mechanisms. This, however, is mitigated by the strict inclusion criteria (primary admission to level-one trauma center, subsequent ICU/ICM care).

In addition, available data are limited to a core dataset, and missing data cannot be accessed. Data concerning co-morbidities, pre-existing medications and frailty status is scarce. Therefore, outcome could not be correlated with prognostic indicators other than age or with frailty scores (i.e. Charleston Comorbidity index or Clinical Frailty Scale). As these play an important role in clinical decision-making, further studies are warranted. This limitation holds also true for (long-term) outcomes and complications, as only mortality and incidence of sepsis are documented.

## Conclusion

Older patients constitute an increasing part of polytraumatized patients and on average are getting older. Outcomes measured by mortality, length of stay and duration of stay on the intensive care unit improve with optimized diagnostic and treatment algorithms. The present data also shows that the majority of patients sustained injuries from falls from low height. We recommend focusing on improving fall prevention and further underline the importance of specialized treatment for older trauma patients. One promising approach is the implementation of specialized trauma centers for older patients, facilitating an interdisciplinary treatment approach from the very beginning.

## Data Availability

The analyzed datasets during the current study are available from the corresponding author on reasonable request.
